# Cefadroxil-Induced Drug Reaction With Eosinophilia and Systemic Symptoms (DRESS) Syndrome After Double Mastectomy for Ductal Carcinoma In Situ: A Case Report

**DOI:** 10.7759/cureus.98209

**Published:** 2025-12-01

**Authors:** Iyawnna Hazzard, Alexandra Hong, Darius J Shahbazi, Shahin Shahbazi

**Affiliations:** 1 Department of Research, College of Medicine, California Northstate University, Elk Grove, USA; 2 Division of Research, College of Osteopathic Medicine, Kansas City University, Kansas City, USA; 3 Department of Hospital Medicine, Kaiser Roseville Medical Center, Sacramento, USA

**Keywords:** cefadroxil, drug reaction with eosinophilia and systemic symptoms (dress), liver injury, rash, viral exanthem

## Abstract

Drug reaction with eosinophilia and systemic symptoms (DRESS) syndrome is a delayed hypersensitivity reaction to medications, characterized by a prodrome of malaise and fever, followed by skin and hematologic manifestations and end-organ damage. Typical causal agents of DRESS syndrome include allopurinol, aromatic anticonvulsants, and antibiotics. However, fewer than 3% of cases suggest cephalosporin-induced DRESS syndrome.

A 43-year-old female developed a diffuse, erythematous, pruritic rash encompassing her whole body after starting prophylactic cefadroxil therapy before a double mastectomy for ductal carcinoma in situ. Her rash continued to worsen despite discontinuation of cefadroxil and developed a fever, myalgias, and generalized weakness. In the emergency department, she was noted to be febrile (103.5 °F) with elevated liver enzymes and eosinophilia, consistent with DRESS syndrome. She was admitted to medicine for further management with systemic steroids, intravenous fluids, and close observation.

DRESS syndrome is a rare manifestation occurring after taking selected drugs. The signs and symptoms of DRESS syndrome may be indolent and persistent even after stopping the offending agent, as seen in this patient. Therefore, prompt treatment is necessary to prevent detrimental complications of the disease.

## Introduction

Drug reaction with eosinophilia and systemic symptoms (DRESS) syndrome is a severe, delayed drug-induced reaction. It is characterized by a prodrome of malaise, pruritus, fever greater than 38.5 °C, followed by skin manifestations, notably a morbilliform rash, facial edema, lymphadenopathy, hematologic manifestations, including atypical lymphocytes and eosinophilia, and end-organ damage involving the liver and kidney [1,2]. This reaction typically manifests two to eight weeks after drug exposure [3].

The true incidence of DRESS syndrome is uncertain due to its varying presentation, dependent on the medication itself and the patient’s immune status, which may result in many undiagnosed cases [1,4]. However, it is estimated that the incidence is greater than 1 case per 10,000 exposures to a medication [1]. One retrospective study found the prevalence of DRESS syndrome to be 2.18 per 100,000 [5].

DRESS syndrome is commonly caused by allopurinol and aromatic anticonvulsants, such as carbamazepine, lamotrigine, and phenobarbital [6]. Certain antibiotics are also a major contributor, especially vancomycin, sulfonamides, and antituberculosis agents, including isoniazid, rifampin, pyrazinamide, and ethambutol. However, cephalosporins were not found to be a significant contributor. One review found the prevalence of cephalosporin-induced DRESS syndrome to be 3.94% [6]. Other common causal agents of DRESS syndrome include dapsone, amitriptyline, hydroxychloroquine, sulfasalazine, omeprazole, and non-steroidal anti-inflammatory drugs [4].

The pathogenesis of DRESS syndrome, otherwise known as drug-induced hypersensitivity syndrome (DIHS), is complex. It is known to be a type IV hypersensitivity reaction in which CD8+ T cells react to structural elements, leading to the production of interferon gamma and tumor necrosis factor alpha and mounting an immune response [4]. These structural elements include, but are not limited to, viral epitopes, drugs, and/or drug metabolites [4]. We present a rare case of a 43-year-old female, one month status post double mastectomy, who promptly developed a diffuse maculopapular body rash, fever, myalgias, and elevated aminotransferases after prophylactic cefadroxil therapy, concerning for DRESS syndrome. The patient gave written informed consent for this case report.

## Case presentation

Presenting concerns

A 43-year-old female with a history of ductal carcinoma in situ (DCIS) who was one month post-op of a bilateral mastectomy presented to the emergency room complaining of a diffuse, pruritic rash affecting her entire body. She previously underwent a prophylactic double mastectomy and bilateral breast reconstruction with microvascular free flap for preventative measures after a biopsy revealed left-sided DCIS. However, this was complicated by a hematoma requiring a revision. She was started on a 30-day course of prophylactic cefadroxil 24 hours before the procedure (day 0). Weeks later, she reported pruritus followed by a pruritic rash on her chest and abdomen. At that time, she had taken only three weeks’ worth of cefadroxil and was prescribed a six-day course of oral and topical prednisone. After one day of taking prednisone, she developed a headache, weakness, malaise, myalgias, and a fever. She denied any recent travel, sick contacts, or any changes in her fragrance or laundry detergent. By the third day of prednisone and 31 days after cefadroxil, the rash spread to her upper and lower extremities, prompting her to come to the emergency department (ED). She denied any nausea, vomiting, diarrhea, urinary symptoms, chest pain, shortness of breath, or cough.

**Figure 1:**
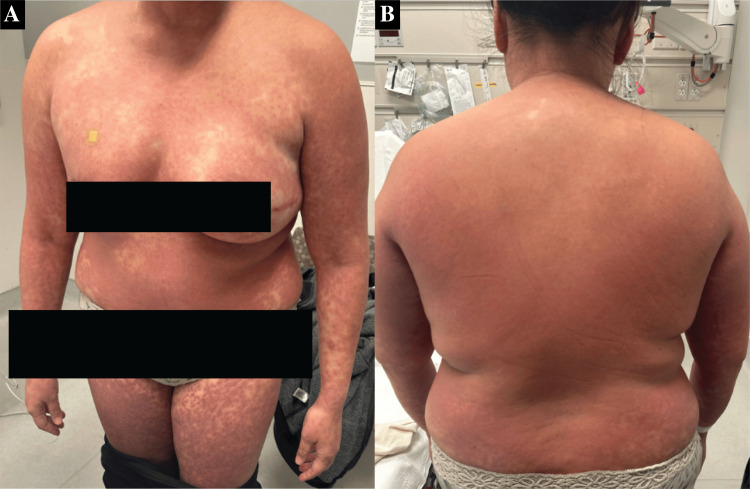
Diffuse erythematous maculopapular rash on the chest, abdomen, and bilateral lower extremities (A), and on the back (B), without ulceration.

In the ED, she was found to be febrile at 103.5 °F (39.72 °C), and physical exam showed a diffuse, erythematous maculopapular rash involving her entire body (Figure 1), including her face.

Laboratory values (Table 1) showed elevated alanine aminotransferase (AST) of 439 IU/L, elevated aspartate aminotransferase (AST) of 259 IU/L, elevated total bilirubin of 2.4 mg/dL, elevated erythrocyte sedimentation rate (ESR) of 29, elevated C-reactive protein (CRP) of 7.4, and low albumin of 3.4. Abdominal ultrasound revealed no cholelithiasis or bile duct dilation. Complete blood count (CBC) revealed a normal white blood cell count (9,100 cells/mL), anemia (hemoglobin 11.0), and an elevated absolute eosinophil count of 8% concerning for DRESS syndrome. A summary of the case timeline is presented in Table 2.

**Table 1:** Laboratory data. Abnormal values are indicated in bold. CBC: complete blood count; BMP: basic metabolic panel; fL: femtoliter; mL: microliter; MCV: mean corpuscular volume; WBC: white blood cell count; mEq: milliequivalents; BUN: blood urea nitrogen; ALT: alanine aminotransferase; AST: aspartate aminotransferase; TSH: thyroid-stimulating hormone; HbA1c: hemoglobin A1c; L: low; H: high

Laboratory	Result	Reference value
Complete blood count (CBC)		
Hemoglobin	11.0 (L)	11.9-14.8 g/dL
Hematocrit	35.1	35%-43%
MCV	88	82.5-98 fL
WBC	9,100	3,800-10,000 cells/mL
Platelets	300,000	153,000-361,000 cells/mL
Neutrophils	48	40%-60%
Lymphocytes	38	20%-40%
Monocytes	5	2%-8%
Eosinophils	8 (H)	1%-4%
Comprehensive Metabolic Panel (CMP)		
Sodium	134 (L)	136-154 mEq/L
Potassium	3.6	3.5-5.0 mEq/L
Chloride	98	98-106 mmol/L
Bicarbonate	26	23-30 mmol/L
BUN	12	8-20 mg/dL
Creatinine	0.58	0.5-1.1 mg/dL
Albumin	3.4 (L)	3.5-5.5 g/dL
Total bilirubin	2.4 (H)	0.3-1.0 mg/dL
ALT	439 (H)	10-40 IU/L
AST	259 (H)	10-40 IU/L
Alkaline phosphatase	328 (H)	30-120 IU/L
Lipase	14	10-140 IU/L
Urinalysis		
pH	5.0	5-9
Specific gravity	1.031 (H)	1.003-1.030
Glucose	Negative	Negative
Ketones	5	Negative
Bilirubin	Small	Negative
Protein	100	Negative
Leukocyte esterase	Negative	Negative
Nitrate	Negative	Negative
Other		
ESR	29 (H)	0-20 mm/hour
CRP	7.4 (H)	≤0.9 mg/dL
Lactate	1.5	≤2 mmol/L

**Table 2:** Case timeline of a 43-year-old female with a history of left-sided ductal carcinoma in situ. All events occurred in the year 2025. ED: emergency department; ALT: alanine aminotransferase; AST: aspartate aminotransferase; ESR: erythrocyte sedimentation rate; CRP: C-reactive protein; US: ultrasound

Day	Event
0	Started on prophylactic cefadroxil 24 hours before surgery.
	Pruritus began.
1	Revision double mastectomy.
	Pruritic rash on chest and abdomen.
24	Persistent pruritic rash on the chest and abdomen.
	Stopped cefadroxil. Started prednisone
28	The patient reported to the ED.
31	Stopped prednisone.
	Rash spread to the face and upper and lower extremities.
35	The patient reported to the ED.
	Exam: diffuse erythematous maculopapular rash involving the entire body.
	ED labs: eosinophils 8%, ALT 439 IU/L, AST 259 IU/L, ESR 29, CRP 7.4
	Abdominal US showed no cholelithiasis or biliary duct dilation.
	Administered lactated ringers, dexamethasone, diphenhydramine, antipyretics, and topical betamethasone.
38	Resolution of fever and eosinophilia and persistent improvement in liver enzymes and pruritus.

Treatment pathway

**Figure 2:**
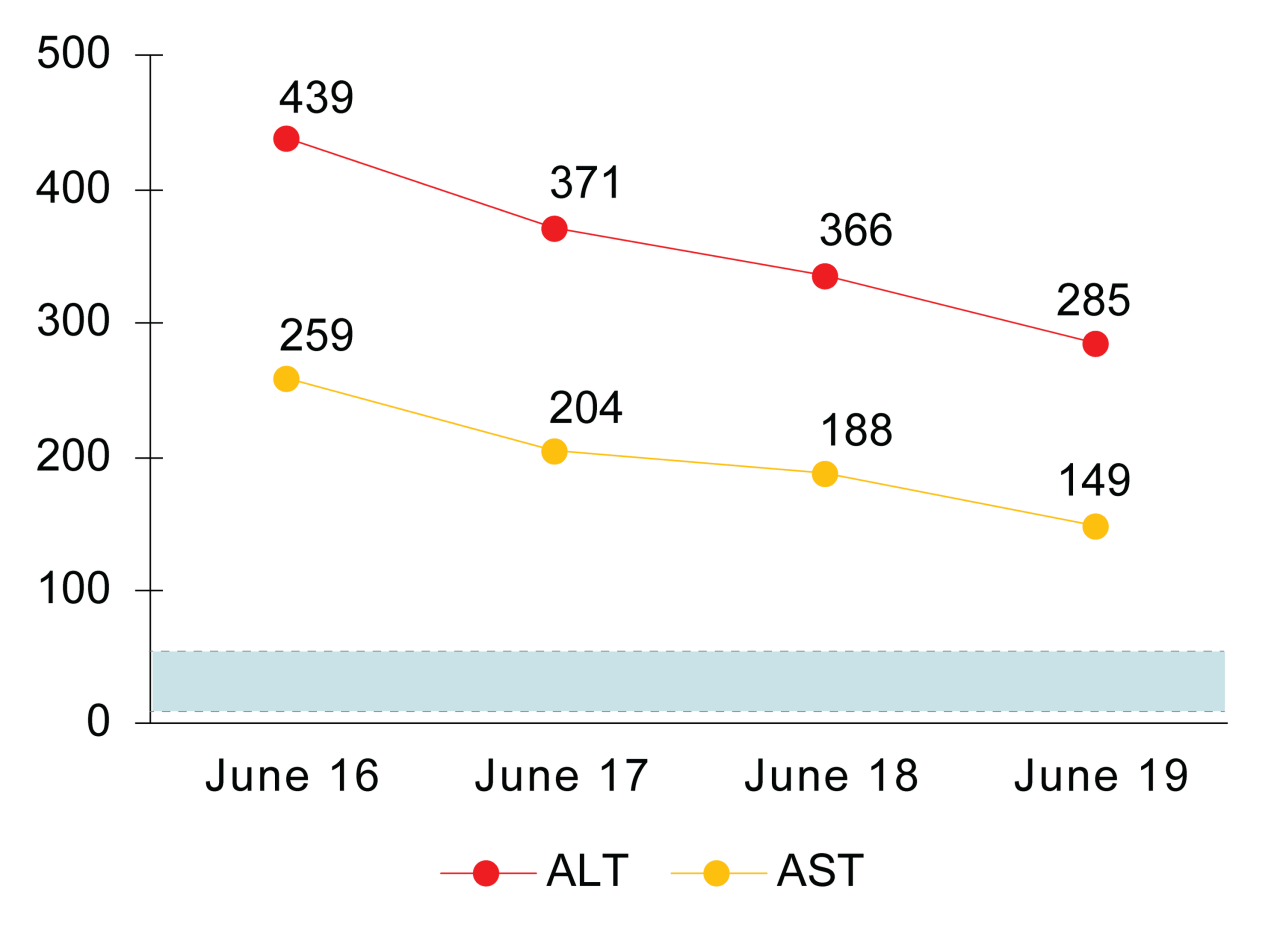
Line graph of liver enzymes after treatment. Y-axis units in IU/L. The blue box indicates the normal range of ALT and AST values. ALT: alanine aminotransferase; AST: aspartate aminotransferase

She was admitted to medicine for further management and observation, pending dermatology, infectious disease, and plastic surgery consultations. In the hospital, she was treated with antipyretics, including oral ibuprofen and acetaminophen, which resolved her fever (97.3 °F). Intravenous (IV) diphenhydramine 25 mg every four hours as needed helped alleviate her pruritus. Given the worsening of the rash, IV steroids were held until dermatology was consulted. DRESS syndrome is typically treated with oral prednisone 0.5-1 mg/kg daily; however, after consultation, oral dexamethasone 10 mg daily was recommended, along with topical betamethasone 0.1% twice daily, due to the presumed exacerbation of the patient’s rash with the initial addition of prednisone. She also received IV hydration with lactated Ringer’s, and daily monitoring of her liver enzymes, CBC, blood urea nitrogen (BUN), and creatinine (Cr) was performed throughout her admission. After therapeutic intervention with IV and topical steroids, IV fluids, antipyretics, and antihistamines, the patient’s liver enzymes demonstrated progressive improvement to normal values (Figure 2).

## Discussion

Differential diagnosis and clinical reasoning

Several differential diagnoses were considered for this case, which included contact dermatitis, viral exanthem such as Epstein-Barr Virus (EBV) infection, bacterial skin and soft-tissue infection (SSTI), acute viral hepatitis, and drug-induced lupus erythematosus (DILE).

Initially, the patient was presumed to have contact dermatitis, given the initial presentation of the rash confined to her torso, and was prescribed steroids. However, this was ruled out given the patient’s lack of history of new detergents and spread of the rash with systemic symptoms. Viral exanthem due to EBV infection commonly presents with fever and a faint or pruritic maculopapular rash [7]; therefore, this was considered a potential diagnosis. She also tested positive for viral capsid antigen (VCA) IgG and EBV nuclear antigen via enzyme-linked immunosorbent assay (ELISA), indicating a previous infection. However, the patient denied symptoms of pharyngitis, sick contacts, and tested negative for IgM EBV VCA via ELISA. Other viral exanthems, such as measles and rubella, were also excluded due to lack of cough and pharyngitis and history of vaccination. The patient’s AST and ALT were 5-10 times the upper limit of normal. Given the patient’s elevated liver enzymes, acute viral hepatitis was also considered, especially considering that ALT > AST is a common finding [8]. However, the patient’s hepatitis B and C panels were negative for acute infection. Infectious disease was consulted for this case and doubted bacterial SSTI despite fever; therefore, antibiotics were held. Plastic surgery was also consulted and ruled out complications of the mastectomy, as her symptoms were systemic and were not solely confined to the breast tissue. Another diagnosis that was considered was DILE. DILE is a lupus-like syndrome due to continuous administration of drugs such as hydralazine, procainamide, isoniazid, calcium-channel blockers, thiazide diuretics, and many more [9] - none of which the patient was taking. DILE commonly presents with myalgias, fever, and skin rashes [9] as seen in our patient. Laboratory studies of DILE reveal elevated EST, positive ANA antibodies, and nearly 95% of patients have positive anti-histone antibodies [9]. Our patient’s autoimmune panel, including anti-dsDNA, anti-Smith, anti-centromere, and anti-chromatin or nucleosomal histone antibodies, was all negative, which did not align with DILE as the diagnosis.

Upon further workup, skin punch biopsy of the right thigh was obtained by dermatology, which revealed patchy basal vacuolar change, rare dyskeratotic keratinocytes, and mild to moderate perivascular lymphohistiocytic infiltrate with few extravasated RBCs and rare eosinophils within the superficial dermis. However, these histological findings, although subtle, are also observed in a viral exanthem; however, DRESS syndrome could not be excluded, given that it is a clinical diagnosis. Therefore, as established through clinical evaluation, the patient’s diffuse pruritic macular rash that persisted over three weeks after cefadroxil, along with her elevated eosinophil count, liver enzymes, and systemic symptoms of fever, myalgias, and generalized weakness, were more consistent with DRESS syndrome.

Previous case reports have also highlighted cephalosporin-induced DRESS syndrome, both involving the third-generation cephalosporin cefotaxime [10,11], with one report confirming reactivity on intradermal testing (IDT) to first-, second-, and third-generation cephalosporins [11]. This indicates cross-reactivity between multiple beta-lactam antibiotics as a structural commonality and further validates the causality of DRESS syndrome as a consequence of cefadroxil.

Follow-up and outcomes

Given the patient's resolution of fever, consistent improvement in liver enzymes, and normal abdominal ultrasound, the patient was discharged home on day 38, with close outpatient follow-up and a five-week oral dexamethasone taper. Topical alclometasone 0.05% twice daily and diphenhydramine as needed were prescribed for the pruritic rash. Some of the late complications of DRESS syndrome include autoimmune thyroiditis or Graves’ disease, diabetes mellitus, and end-organ dysfunction, particularly renal or hepatic failure [12]. The patient’s baseline thyroid-stimulating hormone (TSH) and hemoglobin A1c (HbA1c) were normal. Her baseline BUN and creatinine remained normal throughout her hospital stay; however, her urinalysis showed ketones and protein. Therefore, a three-month follow-up visit was scheduled to further evaluate for long-term sequelae.

## Conclusions

DRESS syndrome is a rare manifestation that occurs after taking selected drugs. We present a rare case of cefadroxil-induced DRESS syndrome while outlining multiple conditions that can mimic its presentation. Such diseases include viral exanthem, DILE, and SSTI. A thorough evaluation and consultation with infectious disease and dermatology aids in prompt diagnosis. The signs and symptoms of DRESS may be indolent and persistent even after stopping the offending agent, as seen in this patient. Finally, DRESS syndrome is known to cause downstream sequelae, particularly endocrinological dysfunction and liver failure. Therefore, prompt treatment, close follow-up, and monitoring are necessary to prevent detrimental complications.
